# Strengthening quality in sexual, reproductive, maternal, and newborn health systems in low- and middle-income countries through midwives and facility mentoring: an integrative review

**DOI:** 10.1186/s12884-023-06027-0

**Published:** 2023-10-05

**Authors:** Rondi Anderson, Sojib Bin Zaman, Abdun Naqib Jimmy, Jonathan M Read, Mark Limmer

**Affiliations:** 1 The Faculty of Health and Medicine, Lancaster University, Lancaster, UK; 2 Department of Health Sciences, James Madison University, Harrisonburg, Virginia USA; 3 Environmental Science Department, Jahangirnagar University, Dhaka, Bangladesh; 4 Lancaster Medical School, Lancaster University, Lancaster, UK

**Keywords:** Midwives, Mentoring, Maternity care, Quality, Health systems strengthening

## Abstract

**Background:**

There is an urgent global call for health systems to strengthen access to quality sexual, reproductive, maternal, newborn and adolescent health, particularly for the most vulnerable. Professional midwives with enabling environments are identified as an important solution. However, a multitude of barriers prevent midwives from fully realizing their potential. Effective interventions to address known barriers and enable midwives and quality sexual, reproductive, maternal, newborn and adolescent health are less well known. This review intends to evaluate the literature on (1) introducing midwives in low- and middle-income countries, and (2) on mentoring as a facilitator to enable midwives and those in midwifery roles to improve sexual, reproductive, maternal, newborn and adolescent health service quality within health systems.

**Methods:**

An integrative systematic literature review was conducted, guided by the Population, Intervention, Comparison, Outcome framework. Articles were reviewed for quality and relevance using the Gough weight-of-evidence framework and themes were identified. A master table categorized articles by Gough score, methodology, country of focus, topic areas, themes, classification of midwives, and mentorship model. The World Health Organization health systems building block framework was applied for data extraction and analysis.

**Results:**

Fifty-three articles were included: 13 were rated as high, 36 as medium, and four as low according to the Gough criteria. Studies that focused on midwives primarily highlighted human resources, governance, and service delivery while those focused on mentoring were more likely to highlight quality services, lifesaving commodities, and health information systems. Midwives whose pre-service education met global standards were found to have more efficacy. The most effective mentoring packages were comprehensive, integrated into existing systems, and involved managers.

**Conclusions:**

Effectively changing sexual, reproductive, maternal, newborn and adolescent health systems is complex. Globally standard midwives and a comprehensive mentoring package show effectiveness in improving service quality and utilization.

**Trial registration:**

The protocol is registered in PROSPERO (CRD42022367657).

## Background

There is an urgent global call for increased availability of quality sexual, reproductive, maternal, newborn and adolescent health (SRMNAH) [1]. Literature finds that professional midwives with enabling environments are an effective solution [2–4]. The global standard for a midwife is delineated by the International Confederation Midwives (ICM). Although most countries are working toward attaining this standard, wide variation in the definition of midwife within countries remains.

Professional midwives have been found to improve quality in both high- and low-resource settings. However, barriers to practice are also noted [5]. Resistance to quality improvement within health systems is one barrier that can require complex system change to address [6]. In spite of clear World Health Organization (WHO) guidance on SRMNAH, there is a perpetuation of low quality–at times harmful–care in low- and middle- income countries (LMICs) [5–7]. The broad sweep of LMICs was chosen to reflect the ubiquitous nature of the problem, however contextual differences between and within countries are acknowledged and warrant further study [7]. The WHO 2021 publication from the Network for Improving Quality of Care for Maternal, Newborn and Child Health (or, the Quality of Care Network) outlines five functions for quality improvement, for LMICs [1]. The first one—onsite support—has been found to be effective in addressing resistance and influencing system change. The provision of onsite support can be key in supporting enabling environments for desired changes, thus it may be needed for midwives to reach their full potential.

Facility mentorship, a type of onsite support, is an increasingly popular approach for enabling quality SRMNAH in LMICs [8, 9]. It comprises both clinical and facility-wide interventions aimed to capacitate and create enabling environments for quality care [10, 11]. Through advocacy, modeling, and problem solving for the needed changes, quality improvements can be achieved.

Mentorship and midwifery have been found to be synergistic as midwives need enabling environments to achieve optimum results, and midwives’ expertise increases the success of mentorship [2, 10, 12, 13]. This review intends to evaluate the bodies of literature on (1) introducing midwives and (2) facility mentoring to better understand facilitators and barriers to implementation of quality, evidence-based SRMNAH care. It aims to provide insight into effective methods of integrating midwives and their related services into health systems [14]. It is hoped that further refinement of our knowledge on this topic will support program efficacy and improve quality of care for the most vulnerable women. The research question was: what is the impact of deploying midwives, and of mentoring midwives, other cadres midwifery roles, managers, and support staff, on providing SRMNAH care in LMICs?

## Methods

An integrative systematic literature review was performed with a narrative synthesis approach [15]. The details of the methodology have been published previously [16]. An initial scoping found abundant literature on midwives in high-resource countries and limited literature focused on LMICs. Very few articles from LMICs had a specific focus on the introduction of ICM-standard midwives, or on mentoring to support newly introduced midwives. For the purposes of this review, the term ‘midwife’ included all skilled health workers providing SRMNAH services and was not limited to midwives meeting ICM standards. Facility mentoring was defined as regular visits to health facilities to support providers, staff, faculty, and or managers. Mentors could engage in observation, guidance, feedback, and/or data collection, all with the intention of improving the quality and availability of SRMNAH services. Supplies and equipment as well as infrastructure support were not considered mentoring. An integrative systematic review was chosen as it encouraged the inclusion of diverse articles, thus allowing for a more robust comprehensive review [15, 17].

### Inclusion and exclusion criteria

The review was guided by the Population, Intervention, Comparison, Outcome (PICO) framework [18]. Inclusion and exclusion criteria are listed in Table 1. The review included literature from the last 13 years (Jan 2010 to May 2023) that addressed systems strengthening in LMICs through the introduction of midwives, enabling environments for midwives, mentoring, and achieving quality of care. Reviews from the past thirteen years are thought to capture current contexts and issues [19]. Only articles published in English were included.

**Table 1:** Inclusion and exclusion criteria

**Inclusion criteria**	**Exclusion criteria**
**• Population**: Midwives, maternity staff, managers, health systems, health facilities, and patients receiving care in LMICs **• Intervention**: The introduction of midwives and/or mentoring **• Comparison**: The differences in efficacy for health system strengthening and quality and availability of services between hospitals with midwives, those without, and those with midwives and mentorship **• Outcome**: Improved quality of care as defined by the presence (or absence) of WHO quality-of-care interventions; improved outcomes; and staff perceptions and experiences of the addition of midwives, mentorship, and transitioning to improved care quality **•** Study design: Any **•** Study type: Qualitative or quantitative **•** Context: LMICs per World Bank definition **•** Publication year after 2010 (2011-2023)	**•** Studies conducted in high-income countries **•** Studies not published in English **•** Studies that delineate plans that have not yet been implemented **•** Studies not focused on the introduction of midwives, mentoring, maternal health systems strengthening **•** Studies that show planning and not impact

*LMICs* low and middle income countries, *WHO* World Health Organization

### Information sources

The review was conducted in February of 2023. The literature was searched through Medline, EMBASE, and CINAHL. In addition to the database search, internet searches of published reports and gray literature, and hand searching of relevant reference lists were performed using a snowball approach. References were managed using an EndNote citation manager.

### Search strategy

The review was carried out using *a priori* planned searches. It was inclusive of all literature that addressed the introduction of midwives and/or the use of mentoring to improve SRMNAH in LMICs, including qualitative experiences of those involved. Predetermined key concepts were searched with specific subject headings and the related Medical Subject Headings (MeSH) or thesaurus terms, as shown in Table 2. The search was ConceptTerms1 AND ConceptTerms2 AND ConceptTerms3. Additionally, we conducted a systematic search of relevant gray literature sources using these search terms and key concepts to include gray literature in this review. After identifying relevant gray literature documents, such as government reports, conference proceedings, and institutional repositories, that align with this research topic, we critically assess their quality and relevance to our research question, applying the PICO inclusion and exclusion criteria. Finally, we synthesized the key findings from the selected gray literature sources alongside findings from peer-reviewed literature.

**Table 2:** Key concepts shaping the literature review and associated search terms

Key concepts	Search terms
Midwives/midwifery	Delivery, Obstetric; maternal health services; midwife* OR midwiv*; maternal; skilled birth attendan*
Supervision/mentoring	Mentoring; mentors; mentor*; supervis*
Care quality/ care improvement	Quality of health care; quality improvement; care quality; outcome; quality improvement; healthcare

*symbol that broadens a search by finding words that start with the same letters

### Study selection

**Fig. 1:**
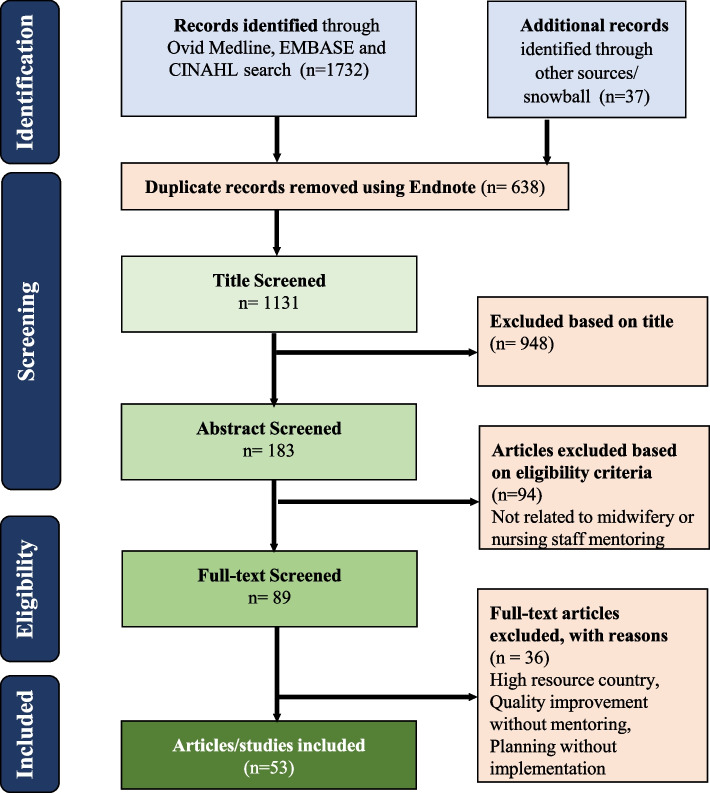
PRISMA flowchart of the literature review

The process of screening and reviewing abstracts and full-text articles based on eligibility criteria is presented in Fig. 1 [20]. After the initial titles were screened, the authors (RA, SBZ, ANJ) screened all abstracts against the inclusion and exclusion criteria. The full texts of all abstracts were then reviewed by the authors (RA, SBZ, ANJ).

### Quality assessment

All selected articles were reviewed for quality and relevance. A combined, modified mixed-methods synthesis tool was used with the Gough (2007) weight-of-evidence framework [21]. The Gough tool guides quality evaluation using four themes: coherence and integrity, appropriateness for answering the question, relevance and focus, and overall assessment (Table 3). Using the tool, each theme was given a rating of high, medium, or low. These ratings then combine to form an overall rank. Author RA and SBZ independently reviewed and ranked the articles based on the above criteria, and results from the individual rankings were discussed. In case of discrepancies in rankings, the final decision was taken by the principal author (RA). All articles were included with recognition given their potential strengths and weaknesses.

**Table 3:** Gough tool for this review

**Dimension**	**Description of review**
A. Coherence and integrity	A generic non-review-specific judgment of the coherence and relevance on its own terms, using the generally accepted criteria for this type of evidence
B. Appropriateness for answering the question	A review-specific judgment about the fitness for the purpose of the evidence for answering the question
C. Relevance and focus	A review-specific judgment about the relevance of the focus of the evidence for the question. This could include issues of propriety in how the research was conducted, which could impact its inclusion and interpretation.
D. Overall assessment	The three above judgments are then combined to give an overall assessment.

Source: Gough ([21], p223)

### Data analysis and presentation

As this was an integrative review, there were multiple types of research used. The articles were sorted by research types using five broad categories: 1) project intervention, 2) retrospective country analysis, 3) qualitative process description, 4) literature review, and 5) modeling study. This helped reduce risk of bias by at once evaluating a wide range of studies and distinguishing findings by their research approach.

A health systems building block framework was used to guide data extraction and analysis by the three authors (RA, SBZ and ANJ). Data from the articles were iteratively compared to identify common sub-themes relevant to the research question [17]. The sub-themes were coded and aggregated to identify emerging themes under the existing health system building blocks: national policies and administration, care quality, health-seeking behaviors, experiences and underlying motivators of staff, health outcomes, access to essential medicine, and information systems were identified (Table 4). Although the themes are distinct, they could also be described as steps in a process, tied to and dependent on each other. They are also aspects of the health systems building blocks.

**Table 4:** Themes and sub-themes

**Themes**	**Sub-themes**
National policies and administration	Gaps between policies and ground realities; allegations of corruption; impact on government spending, professional standardization (or lack of).
Care quality	With mentoring, certain improvements in care provision by maternity staff, quality monitoring systems, and availability of lifesaving medicines and equipment.
Health seeking behaviors	Increased antenatal Care (ANC), increased Skilled Birth Attendance (SBA), increased facility birth, increased immunizations.
Experiences and underlying motivators of midwives and maternity staff	Discomfort related to deployment status and its impact on performance; and providers’, mentors’ and administrators’ appreciation of mentoring.
Health outcomes	Maternal mortality, newborn mortality, improved under five deaths.
Midwifery-led care models	Midwives are lead professionals in delivery of care from pregnancy through postnatal period.

Following theme identification, articles were categorized according to the building blocks. A master table and supplementary tables were created to classify each article according to its Gough score, country of focus, themes, building blocks and methodology. The table also categorized articles according to whether they were inclusive of midwives and or mentoring, if care providers met an international standard for midwives, and how mentoring was defined. To minimize risk of bias in the synthesis of findings, articles involving interventions (methodology type 1) were disaggregated to identify interventions most likely to be transferable. Disaggregation was based on their approaches to measuring outcomes and direction of change. Four outcomes measurement approaches were determined: 1) self-reported or before/after tested knowledge or skills; 2) observed quality improvement at clinical sites; 3) information system health outcome tracking; and 4) facility data on service utilization.

## Results

The results analysis is divided into three sections. We provide an overview of key characteristics, rankings and methodological approaches. We then present an analysis of the two main themes (a) midwives and/or both midwives and mentors and (b) mentoring, which emerged from the studies. Finally, we offer a comprehensive synthesis of the specific findings related to midwifery and mentorship according to the health system building blocks approach observed across the included studies.

Fifty-three articles were included in this review (Fig. 1) [22–73]. Tables 5 and 6 at the end of the article display all article data. Among them, 13 were ranked as high, 36 medium, and four low (Table 5). In addition, 18 focused on the introduction of midwives, 29 on mentoring, and six combined midwife introductions with mentoring. Ten articles described midwives meeting a global ICM standard while the others described a range of categories of staff who were called midwives or were providing maternity care. These included nurses, midwives not meeting ICM standards, midwives with unspecified education, general maternity staff (i.e., not midwives), and skilled birth attendants with unspecified education (Table 7, also at end). Across both midwifery and mentoring articles, theme categories yielded overlap, with all articles aligning with multiple themes.

**Table 5:** Characteristics of the studies included in the review study methods and key findings

Sl.	**First author, country, year**	**Ref**	**Study methods & participants**	**Study type**	**Introduction of midwives**	**ICM- standard midwife**	**Mentoring**	**Manager**	**Outcomes measures**	**Key findings, including barriers and facilitators**
1	S. Tasnim, et al. Bangladesh, 2011	[22]	- Quasi-experimental community trial -10 health facilities were assigned randomly to either intervention (midwives deployed) or controls using only doctors and nurses. -Data were collected from the community members on healthcare uptake.	Intervention evaluation	No	Yes	No	-	Increased service utilization	-Describe the midwifery education process and community mobilization. -As a result of midwives being deployed to health facilities, there has been an increase in the use of ANC, SBAs, and facility delivery.
2	K. Jayanna, et al. Karnataka, India, 2016	[23]	-Partial cluster randomized trial design with two arms, all receiving training and case sheets and one arm receiving mentoring. -Pre- and post-intervention surveys, facility audits, case sheet reviews, and provider interviews.	Intervention evaluation	No	No: Maternity staff unspecified	Yes	Yes	Observed improved quality	-A higher level of drug and supply availability was reported in facilities that received mentoring. -There was improved knowledge among the providers regarding pregnancy and newborn-related complications, providers were more compliant with labour protocols, and the intervention cost was just under 6 USD per delivery.
3	W. Van Lerberghe, et al. Burkina Faso, Cambodia, others, 2014	[24]	Literature review, Ministry of Health sources, and key informant interviews.	Retrospective country analysis	Yes	No: Inclusion of all nurses and unspecified midwives	No	-	Improved outcomes	-To strengthen the health system, it is critical to deploy midwives as defined. -The number of births in SBA and facilities has increased in all socioeconomic quintiles. -There has been a decrease in maternal and neonatal mortality.
4	Singh, et al. Africa, Asia and Latin America/ Caribbean, 2013	[25]	-Recent demographic and health survey data was used to pull data from 9 countries in 3 regions -Logistical regression analysis controlled for data such as socio-economic status, age, parity, education, and region.	Retrospective country analysis	Yes	No: Inclusive of all SBAs as defined by WHO	No	-	Improved outcomes	-Mixed results were observed. -The availability and utilization of SBAs in Latin America and the Caribbean have contributed significantly to reducing maternal and neonatal mortality rates, ensuring safer childbirth, and protecting the health and well-being of mothers and their newborns. -It has been observed in Asia that mothers are not protective for the first day of a child's life, but for the first week.
5	Vieira, et al. Bangladesh, Indonesia, Peru, 2012	[26]	A systematic review	Literature review (SR)	Yes	No: But 4 studies are specific to midwives (not defined as ICM standard)	No	-	Improved outcomes, increased utilization, observed quality	-This review identified six studies that are relevant and of adequate quality. -The introduction of midwives was the subject of four of the studies. -As a result of having midwives closer to the community, the use of SBAs and the rate of caesarean sections increased. -A study found that deploying skilled midwives was associated with reducing maternal mortality.
6	E. Speakman, Afghanistan, 2014	[27]	A review of the policies was triangulated with 8 in-depth interviews with stakeholders	Retrospective country analysis	Yes	No: 18-month training, midwives not ICM standard	No	-	Improved outcomes and increased utilization	-There has been a significant improvement in the SBA and the maternal mortality rate following the introduction of community midwives
7	Z. Mumtaz, et al. Pakistan, 2014	[28]	-Implementation research using an ethnographic approach. -A review of policy and programs was conducted. -Focus groups and interviews were conducted.	Qualitative process review	Yes	No: Midwives, not ICM standard	No	-	No improvements	-There are gaps between the theory of national programs and the reality on the ground. -The importance of verifying assumptions made in program theory in order to improve their implementation. -A lack of motivation among midwives and a lack of acceptance by the community led to a lack of receptiveness. -There has been no significant impact on skilled birth attendance or MMR.
8	S. Webster, et al. Indonesia, 2013	[29]	-A situational overview. -Data were population-based.	Retrospective country analysis	Yes	Yes	No	-	Improved outcomes	-When midwives are employed in public facilities, MMR generally declines; however, midwives leave for private facilities which serve affluent populations and are therefore paid substantially more. -In the absence of midwives, the number of maternal deaths increased. -Midwifery education accelerated rapidly, but it did not improve in quality, and MMR stopped declining.
9	G. Namazzi, et al. Uganda, 2015	[30]	-This study describes changes in 20 health facilities after a mentoring intervention. -Data were gathered by mentors and from government health information systems.	Intervention evaluation	No	No: Midwives unspecified	Yes	Yes	Improved tested knowledge and skill	-A significant improvement in knowledge was observed during testing. -Midwives have demonstrated an improvement in certain skills, according to mentors. -There was an increase in confidence among healthcare providers, including midwives.
10	Fischer EA, et al. Karnataka, India, 2015	[31]	-53 Full- time mentors, 385 rural health centres. -1 visit every two months. -Data gathered through a qualitative inquiry on impact and experience through observations, focus groups, and interviews with involved stakeholders. -Stratified random pre-and-post assessments in selected districts with and without mentoring.	Intervention evaluation	No	No: Maternity staff unspecified	Yes	Yes	Observed improved quality	-The system of care delivery was improved, and healthcare providers reported greater confidence and adherence to guidelines. -Improvement in specific clinical care, including active management of the third stage, decreased inappropriate use of oxytocin, improved immediate newborn care, and improved supply chain management. -To fully develop their clinical competence and confidence, mentors required additional on-the-job support and reinforcement through clinical practice and refresher training.
11	J. Bradley, et al. Karnataka, India, 2017	[32]	-Randomized trial looking at the knowledge and skills of 295 maternity nurses working in 108 rural health centres. -Compared knowledge and skills at baseline and before and after the interventions.	Intervention evaluation	No	No: Nurses	Yes	-	Improved tested knowledge and skill	-Case sheets with mentorship demonstrated significant improvements in both knowledge and skill on common labor room skills, such as newborn resuscitation, prevention of postpartum haemorrhage (PPH), and awareness of signs of an obstetric emergency.
12	R. S. Potty, et al. Karnataka, India, 2019	[33]	-Data were collected by identifying all pregnant women in the predetermined intervention and non-intervention districts	Intervention evaluation	No	No: Maternity staff unspecified	Yes	-	Increased utilization, improved observed improved quality	-As a result of the intervention, there was an increase in the number of ANCs, facility deliveries, length of stay postpartum, and early breastfeeding in the intervention group.
13	P. Schwerdtle, et al. LMICs (Rwanda, Afghanistan, Jordan, Botswana) 2017	[34]	-Systematic review of mentoring programs in LMICs.	Literature review (SR)	No	No: Maternity staff unspecified	Yes	-	Observed improved quality	-Only five studies met the criteria; of these, only one focused on maternity nurses. -It is also included in this review. -All found that mentoring had a positive impact on care quality.
14	Tripathi, Bhamare P, et al. India, 2019	[35]	-Health facilities were assessed using a standardized observation checklist -Before, immediately after, and one year after the intervention, evaluations were conducted.	Intervention evaluation	No	No: Maternity staff unspecified	Yes	-	Improved tested knowledge and skills	-There is no description of the mentorship program. -A significant improvement was observed in all clinical skills, and this improvement was maintained. -In terms of eclampsia management and teaching of danger signs, the best improvements were noted. -Postpartum care and the resuscitation of newborns were found to have the least effect.
15	A. Manzi, et al. Rwanda, 2018	[36]	-A baseline and end-line assessment were conducted at 21 rural health facilities (330 observations) -The study used an observation checklist -Linear mixed-effect regression was used	Intervention evaluation	No	No: Nurses	Yes	Yes	Observed improved quality	-Overall, there was a significant improvement in women who had all danger signs assessed -The results of other ANC assessment items also demonstrated significant improvements. -There was a significant improvement in the quality of ANC visits where nurses monitored vital signs and fetal wellbeing assessment items (fundamental height, heart rate, movement, and position) -The quality of counselling has significantly improved
16	A. Manzi, et al. Rwanda, 2014	[37]	-Mentoring effects on nursing practices before and after	Intervention evaluation	No	No: Nurses	Yes	Yes	Observed improved quality	-Identifying gaps in care and altering systems to address those gaps, such as providing targeted training. -Observations indicate that STI management has improved.
17	G. Tadele Tiruneh, et al. Ethiopia, 2018	[38]	Health facility data: Before and after -Observation, record review, and provider interviews were used to gather data -Regression methods were used to measure the dose-response.	Intervention evaluation	Yes/ No	Yes: Included	Yes	-	Increased service utilization, improved observed quality	-A higher percentage of needs were met, but there were still major gaps, and improved health seeking was observed -There has been a significant increase in the delivery rate of health centres -A dose-response relationship existed between the explanatory variables and the outcome variables. -For every unit increase in EmONC implementation strength score, facility-based deliveries increased by approximately 4.5 percentage points
18	C. Horwood, et al. South Africa, 2019	[39]	-Cross sectional surveys in 39 district hospitals. -Data gathered through an observation checklist. -Health worker knowledge was assessed midpoint and at the endpoint.	Intervention evaluation	No	No: Maternity staff unspecified	Yes	Yes	Observed improved quality and improved knowledge and skills	-Four national mentoring visits over the course of two years associated with accreditation. -An improved level of knowledge among health workers. -The availability of supplies and equipment has improved overall. -Evidence-based care is being provided in greater numbers.
19	Helen Nita Catton, et al. Laos, 2017	[40]	-Community case study describing the implementation of the mentorship program.	Intervention evaluation	Yes	No: Midwives, not ICM - In Laos, the terms SBA and midwife are used interchangeably	Yes	-	Improved tested knowledge	-The steps involved in developing a mentorship are described in detail. -There was a full-time deployment of national mentors to one healthcare facility. -The initial response was positive. -Mentorship was difficult due to a lack of expert mentors in the field. -The assessment framework has not yet been fully developed, but the preliminary results have been promising.
20	S. Rajbhandari, et al. Nepal, 2018	[41]	-Cross-sectional	Intervention evaluation	No	No: Maternity staff unspecified	Yes	-	Improved tested knowledge and skills	-There was mentoring for clinicians and managers in 61 healthcare facilities -A significant improvement in the ability to use partographs and perform normal deliveries.
21	World Bank, et al. Multiple LMICs, 2013	[42]	-Systematic review of impact evaluations: 68 impact evaluations, 33 specifics to SBAs.	Literature review (SR)	Yes	No: Inclusive of all SBAs	No	-	Improved observed quality, improved outcomes	-Some studies have demonstrated that the intervention is linked to an increase in mortality, breastfeeding, and immunization rates in children under the age of five
22	R. Haththotuwa, et al. Sri Lanka, 2012	[43]	-An overview of the mechanisms implemented that have reduced maternal mortality, including midwives.	Retrospective country analysis	Yes	No: Not ICM standard, 18-month training	No	-	Improved outcomes	-Improved MMR was chronologically related to the deployment of community midwives. -A number of interventions were combined with this intervention.
23	G. Taneja, et al. India, 2021	[44]	-A baseline assessment was done using a structured checklist -This was compared with an external endline assessment	Intervention evaluation	No	No: Not ICM standard	Yes	Yes	Observed improved quality	-An integrated approach was effective in reducing maternal and newborn mortality. -The approach demonstrated a successful operational design for improving the delivery of intrapartum and immediate postpartum care.
24	Dale A. Barnhart, et al. India, 2019	[45]	-A coaching intensity was assigned to each birth, and health outcomes were assessed using regression models.	Intervention evaluation	No	No: Not ICM standard	Yes	Yes	Observed improved quality	-A coaching program was associated with improvements in care quality -Higher levels of adherence were associated with a higher frequency of coaching -It was found that the interventions associated with health outcomes were not sustained
25	Geldsetzer P, E. Larson, et al. Tanzania, 2019	[46]	-Cluster-randomized control study	Intervention evaluation	No	No: Clinical officers, nurses, medical attendants	Yes	-	Observed improved quality	-In primary care clinics with adequate infrastructure and higher competence of service providers, quality improvement interventions are effective. -It is essential to improve the quality of pre-service education in order to produce competent and ethical employees
26	Ngabonzima, et al. Rwanda, 2020	[47]	-Mentorship model was designed based on interprofessional collaboration. -Field visits were conducted in 10 hospitals by 5-person mentor teams over 3 years.	Intervention evaluation	No	Yes	Yes	Yes	NA	-Mentees were pleased with the mentorship program, which identified near misses and saved lives during mentorship visits. -The implementation of a mentorship model based on interprofessional collaboration, as well as the involvement of the hospital's leadership, produces positive results
27	Ngabonzima, et al. Rwanda, 2021	[48]	-Mentors were trained and conducted monthly visits to 111 health centres. -Mentees began coaching other colleagues after 6 months.	Intervention evaluation	No	Yes	Yes	Yes	Improved tested knowledge and skill	-Onsite mentoring resulted in equal training of health care providers, as well as improve their knowledge and self-efficacy
28	L. Haskins, et al. South Africa, 2020	[49]	-27 clinics were evaluated. -The intervention was implemented sequentially -Five waves of data collection were carried out. -Data were analyzed using a multi-level, mixed-effect logistic regression.	Intervention evaluation	No	No care is provided by a team of health workers	Yes	Yes	Observed improved quality	-The coverage of some services has been significantly improved -The QI intervention did not achieve the substantial changes that are required to provide comprehensive services to all mothers and children. -Policy changes were necessary to achieve some of the mentoring goals
29	Stephens, et al. Tanzania, 2019	[50]	-A multi-faceted approach that included training and mentorship for healthcare providers was used over a 30-month period. -PAC service statistics and mentorship observation data were analyzed.	Intervention evaluation	No	No	Yes	Yes	Increased utilization	-An increase in postabortion care (PAC) and contraceptive uptake was found to be significant. -A significant improvement in the quality of service was observed among mentored providers, with slight variation between nurses and doctors. -A PAC client mentoring program has resulted in improvements in provider capacity to provide postabortion clients with voluntary contraceptive services.
30	Delaney, et al. India, 2021	[51]	-Secondary analysis of cluster-randomized trials that included women giving birth at 30 primary and community health facilities.	Intervention evaluation	No	No	Yes	-	Observed improved quality	-After the intervention, oxytocin use was successfully reduced and sustained for four months -Oxytocin was administered more frequently after delivery to prevent postpartum haemorrhages following the intervention
31	Magge, et al. Rwanda, 2020	[52]	-A pre-post intervention study measured change in the maternal and newborn quality of care and neonatal mortality using facility register data.	Intervention evaluation	No	Yes	Yes	Yes	Improved outcomes, observed improved quality	-Improvements were observed in facility readiness, quality of care, and neonatal mortality. -In the rest of Rwanda, neonatal mortality did not decline. -The level of satisfaction among patients remained the same
32	Hoover, et al. India, Rwanda, Zambia, South Africa, 2020	[53]	-Literature review of nurse mentoring research from six databases.	Literature review	No	No	Yes	-	NA	-Nurse mentoring in the workplace can serve as a viable avenue for improving the role of nurses and midwives in strengthening people-centered health systems. -To make mentoring models more transparent and transferrable, future research should analyze key features of programs.
33	McFadden, India, 2020	[54]	-Literature review of 15 databases	Literature review	No	No	Yes	Yes	NA	-Midwives were restricted in their scope of practice due to a lack of competency and lack of legislation recognizing midwives as autonomous professionals. -Facilitators included monitoring and supervising staff, utilizing midwives' full scope of practice with good referral systems, and enhancing the experience of women receiving maternity care
34	Rao, et al. India, 2019	[55]	-Quasi-experimental post-test with a matched comparison group. -Analysis was based on 335 ANMs (237 mentored and 98 comparisons) and 42 staff nurses (28 mentored and 14 comparisons).	Intervention evaluation	No	No -ANMs in India	Yes	-	Observed improved quality	-Through mentoring, Axillary Nurse Midwives (ANMs) were able to take corrective actions to manage normal deliveries by 17.5%, postpartum hemorrhages by 25.9%, and neonatal resuscitation by 28.4%. -Nurses with mentorship and staff nurses with no mentorship showed no significant difference in average abilities. -ANMs and nurses were only able to perform actions correctly about half of the time. -Following the completion of mentoring, the ability of ANM declined.
35	Waiswa, et al. Uganda, 2021	[56]	-Program documentation; -The program was implemented in 3 phases (inception, training, and collaborative).	Intervention evaluation	No	No	Yes	Yes	Improved outcomes	-A consistent decline in maternal and newborn mortality was demonstrated during the collaborative hospital phase as a result of mentorship. -The decline occurred without significant changes in the number of deliveries or caesarean sections. -Mentorship of nurses resulted in better outcomes for pregnant women and newborns, as well as the institutionalization and scaling up of quality improvement processes
36	Osthuizen, et al. South Africa, 2019	[57]	-A mixed-method intervention study -Perinatal outcome indicators for the year before the intervention, during, and year after the intervention were compared	Intervention evaluation	No	Yes	Yes	-	Improved outcomes	-The number of in-facility fresh stillbirths, meconium aspiration, and birth asphyxia decreased significantly -Control group members also showed some decline during the study period due to the support of district clinical specialist team members.
37	Namazzi, et al. Kenya, Uganda, 2021	[69]	-Implementation of an integrated package of interventions, including mentoring	Intervention evaluation	No	No	Yes	Yes	Improved outcomes	-A 34% reduction was found in the combined odds of a fresh stillbirth and neonatal mortality.
38	Feeley, et al. LMICs, 2021	[58]	-Mixed method systematic review in five databases with a mixed method convergent synthesis.	Literature review (SR)	No	No	Yes	Yes	Tested knowledge and skill	-It was found that practitioners required and welcomed additional specific training in this area, in which a variety of teaching methods were used. -To develop competency and expertise, clinical mentorship and observation of others confidently using a wide range of instruments were also required. -Concerns regarding poor outcomes and litigation were also raised.
39	Schuldt, et al. LMICs, 2019	[59]	-An integrative literature review	Literature review	Yes	Yes	No	Yes	NA	-Midwifery-led care is provided across low- and middle-income countries, but the lack of enabling factors limits the quality of this care. -Midwives reported feeling unsupported by their managers
40	Anderson R (urban hospital), et al. Bangladesh, 2022	[60]	- Introduction of midwives with mentoring in two urban (medical college) hospitals -Data collected over time on improvements in care quality. -Statistical significance test done over time	Intervention evaluation	Yes	Yes	Yes	-	Observed improved quality	-Quality improved with the introduction of midwives and mentors, and the quality improvement increased over time. WHO routine quality interventions were noted.
41	Anderson R (rural hospital), et al. Bangladesh, 2022	[74]	-Mixed-methods observational study -Upazila Health Complexes (rural facilities) -Logistic regression	Intervention evaluation	Yes	Yes	Yes	Yes	Observed improved quality	- Midwives did improve quality and they did so more with mentoring, Midwives meeting resistance from managers to practice to their full scope including managing emergencies.
42	Malin Bogren, et al. Bangladesh, 2017	[61]	-It is a descriptive report -Case study	Qualitative process review	Yes	Yes	No	-	NA	- Described the early experiences of introducing midwives to Bangladesh
43	Bogren, Malin, et al. Nepal, 2013	[62]	-33 semi-structured interviews with different stakeholders -Data were analyzed using content analysis.	Qualitative process review	Yes	Yes	No	-	NA	-Global and national standards contributed to the establishment of midwifery associations by bringing together professionals and stakeholders. -There were several challenges for both countries, including national commitments without a complete set of supporting documents, a lack of professional recognition, and competing interests, views, and priorities.
44	Petra ten Hoope-Bender, et al. 2014	[63]	-Analyses of various studies -Modelling of deaths averted	Retrospective country analysis	Yes	No	No	-	Increased service utilization	-Expanded coverage of the SRMNAH services -Strengthening the midwifery profession through policy recommendations
45	Save the children, Bangladesh, 2022	[64]	-Descriptive report of the outcomes of introducing mentors where midwives had recently been deployed	Intervention evaluation	Yes	Yes	Yes	Yes	Observed improved quality and increased service utilization	-Midwives have the potential to play a significant role in institutionalizing evidence-based MNC and reducing unnecessary and sometimes harmful interventions. -Evidence-based MNH practices demonstrate significant improvements in the quality of care -Midwives and other service providers' competencies improved rapidly due to education site compliance with the curriculum.
46	Sangy MT, et al. Multiple LMICs, 2023	[70]	- A systematic review, utilizing mixed-methods, to analyze primary research studies that captured the perspectives of the implementation of midwife-led care in LMICs.	Literature review (SR)	Yes	No, all in midwife’s roles but no ICM standard	No	Yes	NA	-Increasing awareness and confidence through home visits, meetings, social networking, and campaigns boosts the adoption of midwife-led care in LMICs. - Adapting midwifery education to global standards (ICM) and LMIC-specific needs ensures contextualized care delivery.
47	Erlandsson K, et al. Bangladesh, 2018	[65]	-A process evaluation of a mentorship program	Intervention evaluation	Yes	No	Yes	Yes	Self-reported or tested improved knowledge of skill	-Faculty members expressed their appreciation and provided examples of how they had changed or improved their practice. -The barriers to participation included a lack of English proficiency and the inability to visit all facilities.
48	Bogren, et al. Bangladesh, 2018	[66]	Focus group with midwifery students	Qualitative process review	Yes	Yes	No	Yes	NA	-As per midwives’ students, there are a number of professional barriers that they perceive within the health system, such as heavy workloads and a shortage of staff who are not utilized to their full capacity within the health system. -A lack of recognition in the medical hierarchy resulted in midwives having limited autonomy. -Supply shortages, low salaries earned by midwives, and housing and transportation insecurity represent economic barriers.
49	Bartlett L, et al. Total 58 LMICs, 2014	[67]	-Comprehensive/ list modelling	Modelling study	Yes	No	No	No	NA	-The cost-effectiveness of increasing midwifery and obstetrics is greater than the cost-effectiveness of increasing obstetrics alone -The midwifery model was almost twice as cost-effective as the obstetrics model when family planning was considered
50	Dawson AJ, et al. 2015	[68]	-A systematic review -Eleven papers described research in LMIC contexts	Literature review (SR)	No	No	Yes	Yes	Improved outcomes	-The review found that when nurses and midwives collaborated with other health providers, organizations, and communities, it led to better healthcare and outcomes. -This review emphasizes the significance of a conceptual framework to plan leadership and governance approaches, management strategies, and education and training efforts for scaling up nurses and midwives' roles. This can help enhance access to primary healthcare for vulnerable populations.
51	Mwansisya T, et al. Tanzania, 2022	[71]	-Quasi-experimental design with pre-and post-intervention evaluation -The baseline was compared with two endline groups: those with intervention (training and onsite mentorship) and those without.	Intervention evaluation	No	No	Yes	-	self-reported and tested knowledge and skill	- A comparison between groups before and after intervention revealed statistically significant differences in all the dimensions of the self-reported SRMNAH performance in scores. -Comparisons between intervention and control groups revealed statistically significant differences in intraoperative care, leadership skills, and EmONC.
52	Pappu NI, et al. Bangladesh, 2023	[72]	-Semi-structured interview with 55 midwives, midwifery educators and final year midwifery students -Provided an online MSc program educational intervention in midwifery -Qualitative content analysis	Qualitative process review	Yes	Yes	No	-	NA	-To sustain the implementation of midwifery centres in Bangladesh, it is essential to strengthening the foundations of their care model. This requires addressing five identified issues: 1) Limited community access, 2) Inadequate focus on acceptable care standards, 3) Weakness in providing respectful, woman-centered care, 4) Insufficient community engagement, and 5) Lack of integration with the healthcare system.
53	BRAC, Bangladesh, 2022	[73]	-Case study or program description. -No intervention. -It is a description of barriers and facilitators and success and challenge of introducing midwives including midwifery education.	Retrospective country analysis	Yes	Yes	No	No	Improved quality	-Midwifery education was established and quality improvements were made. -Midwives were introduces close to communities and monitoring found improved quality interventions

*EmONC* emergency obstetric and newborn care, *SRMNAH* sexual, reproductive, maternal, newborn and adolescent health Manager (i.e. mentoring of the existing managers, principal, local authority or QI), *PHC* primary health care complex

**Table 6:** Categorization of the selected articles according to quality rank and WHO building blocks approaches

Sl.	**First author, country, year**	**Ref**	**Quality rank**	**Endline, baseline and after study**	**Leadership and governance**	**Health service delivery**			**Health workforce**	**Access to medicine and supplies**	**Data/ health information system**	**Finance**	**Experiences & motivators**
						**Health seeking**	**Care quality**	**Health outcomes**					
1	S. Tasnim, et al. Bangladesh, 2011	[22]	HHH (H)	Y	-	Y	-	-	-	-	-	-	Y
2	K. Jayanna, et al. Karnataka, India, 2016	[23]	HHH (H)	Y	Y	-	Y	-	-	Y	Y	Y	Y
3	W. Van Lerberghe, et al. LMICs, 2014	[24]	MMM (M)	-	Y	Y	-	-	-	-	-	Y	-
4	Singh, et al. LMICs, 2013	[25]	MHM (M)	-	-	-	-	Y	-	-	-	-	-
5	Vieira, et al. Bangladesh, Indonesia, Peru, 2012	[26]	HMM (M)	-	Y	-	-	Y	-	-	-	Y	-
6	E. Speakman, Afghanistan, 2014	[27]	LHL (M)	-	Y	-	-	Y	-	-	-	Y	-
7	Z. Mumtaz, et al. Pakistan, 2014	[28]	HHM (H)	-	Y	-	-	Y	-	-	-	Y	Y
8	S. Webster, et al. Indonesia, 2013	[29]	LHL (L)	-	Y	-	-	Y	-	-	-	Y	Y
9	G. Namazzi, et al. Uganda, 2015	[30]	LLM (M)	-	Y	-	-	Y	-	Y	Y	-	Y
10	Fischer EA, et al., India, 2015	[31]	MLM (M)	Y	-	-	-	Y	-	Y	Y	-	-
11	J. Bradley, et al. Karnataka, India, 2017	[32]	HMM (M)	-	-	-	-	Y	-	-	Y	-	-
12	R. S. Potty, et al. India, 2019	[33]	HML (M)	-	-	-	-	Y	-	-	-	-	-
13	P. Schwerdtle, et al. LMICs 2017	[34]	HMM (M)	-	-	Y	Y	-	-	-	-	-	-
14	Tripathi, Bhamare P, et al. India, 2019	[35]	MHM (M)	Y, and one year following	-	-	Y	-	-	-	-	-	-
15	A. Manzi, et al. Rwanda, 2018	[36]	HMH (H)	-	Y	-	Y	-	-	-	Y	-	-
16	A. Manzi, et al. Rwanda, 2014	[37]	LMM (M)	Y	Y	-	Y	-	-	-	Y	-	-
17	G. Tadele Tiruneh, et al. Ethiopia, 2018	[38]	HML (M)	-	-	Y	Y	-	-	-	-	-	-
18	C. Horwood, et al. South Africa, 2019	[39]	MML (M)	-	Y	-	Y	-	-	Y	-	-	-
19	Helen Nita Catton, et al. Laos, 2017	[40]	MLH (M)	-	-	-	Y	-	-	-	-	-	-
20	S. Rajbhandari, et al. Nepal, 2018	[41]	MMM (M)	-	-	-	Y	-	-	-	-	-	-
21	World Bank, et al. Multiple LMICs, 2013	56 [42]	MLL (L)	-	-	Y	Y	-	-	-	-	-	-
22	R. Haththotuwa, et al. Sri Lanka, 2012	[43]	MLL (L)	-	Y	-	-	Y	-	-	-	Y	-
23	G. Taneja, et al. India, 2021	[44]	MHM (M)	Y	Y	-	Y	-	-	Y	-	-	-
24	Dale A. Barnhart, et al. India, 2019	[45]	MHH (H)	-	Y	-	Y	-	-	Y	-	-	-
25	Geldsetzer P, E. Larson, et al. Tanzania, 2019	[46]	HML (M)	Y	-	-	Y	Y	-	-	-	-	-
26	Ngabonzima, et al. Rwanda, 2020	[47]	MHH (H)	-	Y	-	Y	-	-	-	-	-	Y
27	Ngabonzima, et al. Rwanda, 2021	[48]	MLL (L)	-	Y	-	Y	-	-	-	-	-	Y
28	L. Haskins, et al. South Africa, 2020	[49]	HMM (M)	-	Y	-	Y	-	-	Y	-	-	-
29	Stephens, et al. Tanzania, 2019	[50]	MMM (M)	-	Y	Y	Y	-	-	-	-	-	-
30	Delaney, et al. India, 2021	[51]	HHM (H)	-	-	-	Y	-	-	-	Y	-	-
31	Magge, et al. Rwanda, 2020	[52]	HHM (H)	-	Y	-	Y	Y	-	Y	-	-	-
32	Hoover, et al. LMICs, 2020	[53]	HML (M)	-	-	-	Y	-	-	-	-	-	-
33	McFadden, India, 2020	[54]	HMM (M)	-	Y	-	Y	-	-	-	-	-	-
34	Rao, et al. India, 2019	[55]	MHH (H)	Y	-	-	-	-	-	-	-	-	-
35	Waiswa, et al. Uganda, 2021	[56]	MML (M)	Y	-	-	-	-	-	-	Y	-	-
36	Osthuizen, et al. South Africa, 2019	[57]	MML (M)	Y	-	-	-	-	-	-	-	-	-
37	Namazzi, et al. Kenya, Uganda, 2021	[69]	HML (M)	Y	-	-	-	-	-	-	Y	Y	-
38	Feeley, et al. Ecuador, South Africa, India, 2021	[58]	HMM (M)	Y	Y	-	Y	-	-	-	Y		Y
39	Schuldt, et al. LMICs, 2019	[59]	HHL (M)	-	Y	-	Y	Y	-	-	-	-	Y
40	Anderson R (urban), et al. Bangladesh, 2022	[60]	HHH (H)	-	-	-	Y	-	-	-	-	-	-
41	Anderson R (rural), et al. Bangladesh, 2022	[74]	HHH (H)	-	Y	Y	-	-	-	Y	-	-	Y
42	Malin Bogren, et al. Bangladesh, 2017	[61]	MHM (M)	-	Y	-	-	-	-	-	-	-	-
43	Bogren, Malin, et al. Nepal, 2013	[62]	MML (M)	-	Y	-	-	-	-	-	-	-	-
44	Petra ten Hoope-Bender, et al. 2014	[63]	MMM (M)	-	Y	-	-	-	-	-	-	-	-
45	Save the children, Bangladesh, 2022	[64]	MHL (M)	Y	Y	Y	Y	-	-	Y	-	-	-
46	Sangy MT, et al. Multiple LMICs, 2023	[70]	HMM (M)	-	Y	-	-	-	-	-	-	-	-
47	Erlandsson K, et al. Bangladesh, 2018	[65]	MMM (M)	-	Y	-	Y		-	-	-	-	-
48	Bogren, et al. Bangladesh, 2018	[66]	MMM (M)	-	Y	-	Y		-	-	-	-	-
49	Bartlett L, et al. Total 58 LMICs, 2014	[67]	HHM (H)	-	-	-	-	-	-	-	-	Y	-
50	Dawson AJ, et al. 2015	[68]	MMM (M)	-	Y	-	Y	-	Y	Y	-	Y	-
51	Mwansisya T, et al. Tanzania, 2022	[71]	HMM (M)	Y	-	-	Y	-	-	-	-	-	-
52	Pappu NI, et al. Bangladesh, 2023	[72]	HHM (H)	-	Y	-	-	-	Y	-	-	-	-
53	BRAC, Bangladesh, 2022	[73]	LHM (M)	-	-	Y	Y	-	Y	-	-	-	-

*H* high, *M* medium, *L* low, *LMIC* low- and middle-income country

**Table 7:** Articles by provider type

**Provider type**	**Total**	**Date range**	**Article list**
ICM standard midwife	**10**	2011- 2023	S. Tasnim, Bangladesh 2011
			G. Tiruneh, Ethiopia 2018
			S. Webster, Indonesia 2013
			Ngabonzima, Rwanda, 2021
			Magge, Rwanda, 2020
			Schuldt, Africa, Eastern Mediterranean, South-East Asia, Latin America, 2019
			Ngabonzima, Rwanda, 2020
			Anderson R, Bangladesh, 2022
			BRAC, Bangladesh, 2022
			Pappu NI, et al. Bangladesh, 2023
Unspecified midwives and or non ICM midwives	**15**	2012-2021	Z. Mumtaz, Pakistan 2014
			Vieira, Bangladesh, Indonesia, Peru 2012
			E. Speakman, Afghanistan 2014
			G. Namazzi, Uganda 2015
			H. Catton, Laos 2017
			R. Haththotuwa, Sri Lanka 2012
			Dalehart, India, 2019
			Stephens, Tanzania, 2019
			McFadden, India, 2020
			Rao, India, 2019
			Horwood, South Africa, 2019
			Feeley, Ecuador, South Africa, India 2021
			Waiswa, Uganda, 2021
			Osthuizen, South Africa, 2019
			Namazzi, Kenya, Uganda, 2021
Unspecified SBA and mixed nurses and midwives	**3**	2013-2014	Singh, Africa, Asia and Latin America / Caribbean 2013
			W. Van Lerberghe, Burkina Faso, Cambodia, Morocco, Indonesia 2014
			World Bank, Multiple LMICs 2013
Nurses	**12**	2013-2021	A. Manzi, Rwanda, 2018
			J. Bradley, India, 2017
			A. Manzi, Rwanda, 2013
			Dalehart, India, 2019
			Ngabonzima, Rwanda, 2021
			L. Haskins, South Africa, 2020
			Stephens, Tanzania, 2019
			Hoover, India, Rwanda, Zambia, South Africa, 2020
			McFadden, India, 2020
			Rao, India, 2019
			Namazzi, Kenya, Uganda, 2021
			Ngabonzima, Rwanda, 2020
Unspecified maternity staff	**13**	2015-2022	K. Jayanna, India 2016
			P. Schwerdtle, LMICs 2017
			R. S. Potty, India 2019
			P. Bhamare, India 2018
			S. Rajbhandari, Nepal 2018,
			C. Horwood, South Africa 2019
			A. Fischer, India 2015
			Akuze, Uganda, Benine, Malawai, Tanzania, 2021
			G. Taneja, India, 2021
			E. Larson, Tanzania, 2019
			Delaney, India, 2021
			Magge, Rwanda, 2021
			Feeley, Ecuador, South Africa, India 2021

*ICM* International Confederation of Midwives, *SBA* skilled birth attendants

Among the articles focused on midwives and/or both midwives and mentors, seven assessed interventions, seven were retrospective country analyses, five were qualitative process descriptions, four were literature reviews, and one was a modeling study (Table 5). Key barriers were found across articles that limit midwives’ ability to provide quality care [2, 12, 39, 66, 74]. These included policies and work environments that relegated midwives to support roles and constrained their scope and opportunities for growth. For instance, midwives were found to have limited opportunities to attend normal births, and restrictions from managers on providing aspects of evidence-based care, including managing life threatening emergencies. In her integrative review of midwifery programs in LMICs, Schuldt (2019) noted that only one third of midwives were practicing to their full scope [12]. Despite the challenges, all midwives were providing aspects of midwifery care and many midwives were successfully expanding their roles and improving care quality [36, 59, 64, 73]. Evidence-based policy and guidelines, supportive management, mentoring, and continuous professional development all enabled midwives’ performance. Practicing to their full competencies was more likely when midwives met a global standard and when facility mentoring was provided [59, 60, 64, 73].

Of the articles that addressed mentoring (including mentoring of midwives), thirty evaluated mentoring interventions and five were literature reviews. Most mentors were project-based, providing support to government health facilities; some were government employees [23, 36, 40, 44, 45, 61, 62]. Except for one project that used international mentors, all mentors were national nurses, midwives, or doctors and all mentorship was conducted onsite [64, 65]. Mentors largely received pre-mentoring training of up to a week, while some described only existing professional expertise. Three studies described a five week training [23, 31–33, 48]. Frequency of mentor visits and mentorship approaches varied across projects. Most mentors conducted from bi-weekly to bi-monthly visits ranging from a total of six facility visits to bi-weekly visits for over 18 months [27, 43, 48, 52]. In two projects, mentors were deployed full time [69, 74]. Most mentoring visits lasted one day and visit frequency was positively correlated with quality improvement [45]. While most mentoring focused on service delivery, three articles described mentoring as part of midwifery education to improve quality of classroom teaching and clinical teaching at practice sites [60, 64–66]. Components of mentoring visits included group teaching, case studies, bedside teaching, assessing and advocating for supplies and equipment, establishing and reinforcing data systems, and providing problem solving support [23, 30, 36, 40, 44, 45, 47, 61, 62]. Checklists for mentors to guide their mentoring were mentioned in ten studies [49–51, 58, 69]. The mentoring programs that found improvements in outcomes mentored at least twice per month for at least a 3-month duration [49, 51].

The six studies that described introducing midwives in education and practice *with* mentoring support found additional benefits of mentoring when introducing a new midwifery profession [32–37]. This was largely because of improved enabling environments. The benefits of combining mentoring with the deployment/strengthening of midwives include improved quality of care and improved service utilization. One study found statistically significant improvements in use of ANC cards and partographs over what was found with introducing midwives alone [60].

Across all 31 articles that described interventions, none achieved 100% of identified quality improvement goals. Nine articles highlighted gaps in achieving desired goals [37, 38, 41, 43, 46, 47, 69–71]. Outcomes measurement approaches with direction of change are delineated in Table 7. Participant self-reported or researcher tested improvements in knowledge or skill, and researcher observed quality improvements were the most common approaches. Twenty-seven and 20 articles respectively showed improvements in outcomes using these approaches. Eleven articles reported improved outcomes using health facility data, eight reported increased service utilization, nine highlighted gaps in achieving desired goals, and one reported no improvement. Most intervention articles included baseline and endline observations and some were retrospective evaluations looking at sustainability. Notably, due to larger numbers of mentorship articles examining interventions, more rigorous outcomes measurement information is available on mentoring than on introducing midwives.

Across all articles, the most common building block themes were governance and leadership, and service delivery, with 31 and 28 articles aligning respectively (Table 6). Eleven or fewer articles aligned with access to essential medicine and supplies, data/ health information systems, finance, and health workforce. Studies examined 23 countries: Afghanistan, Bangladesh, Benin, Botswana, Burkina Faso, Cambodia, Ethiopia, India, Indonesia, Jordan, Kenya, Laos, Morocco, Malawi, Nepal, Pakistan, Peru, Rwanda, South Africa, Sri Lanka, Tanzania, Uganda and Zambia (Table 8) [33, 37, 39–41, 43, 44, 47, 52, 58, 62, 64, 65, 69–71, 73–75]. The following sections discuss the findings in further detail according to the health system building blocks, with midwifery and mentorship specific findings presented separately within each section.

**Table 8:** Categorization of the selected articles according to region and country

**Region**	**Countries**	**Date range**	**Article list**
**Africa**	South Africa	2019-2020	C. Horwood, South Africa, 2019
			L. Haskins, South Africa, 2020
			Oosthuizen, South Africa, 2019
	Ethiopia	2018	G. Tiruneh, Ethiopia, 2018
	Rwanda	2013-2021	A. Manzi, Rwanda, 2018
			P. Schwerdtle, LMICs, 2017
			A. Manzi, Rwanda, 2013
			Ngabonzima, Rwanda, 2021
			Magge, Rwanda, 2021
			Hoover, India, Rwanda, Zambia, South Africa, 2020
			Ngabonzima, Rwanda, 2020
	Uganda	2015-2021	G. Namazzi, Uganda 2015
			Akuze, Uganda, Benine, Malawai, Tanzania, 2021
			Waiswa, Uganda, 2021
			Namazzi, Kenya, Uganda, 2021
	Botswana	2017	P. Schwerdtle, LMICs, 2017
	Burkina Faso	2014	W. Van Lerberghe, Burkina Faso, Cambodia, 2014
	Benine, Malawi	2021	Akuze, Uganda, Benine, Malawai, Tanzania, 2021
	Tanzania	2019	E. Larson, Tanzania, 2019
			Stephens, Tanzania, 2019
	Kenya	2021	Namazzi, Kenya, Uganda, 2021
**South and East Asia**	Bangladesh	2011-2023	S. Tasnim, Bangladesh, 2011
			Anderson R, Bangladesh, 2022
			Malin Bogren, Bangladesh, 2017
			Erlandsson K, Bangladesh, 2018
			Save the children, Bangladesh, 2022
			Bogren, Bangladesh, 2018
			Anderson R (2), Bangladesh, 2022
			Vieira, Bangladesh, Indonesia, Peru, 2012 Pappu NI, et al. Bangladesh, 2023
	Sri Lanka	2012	R. Haththotuwa, Sri Lanka, 2012
	India	2015-2021	K. Jayanna, 2016
			J. Bradley, India, 2017
			R. S. Potty, India, 2019
			P. Bhamare, India, 2018
			A. Fischer, India, 2015
			G. Taneja, India, 2021
			Dalehart, India, 2019
			Delaney, India, 2021
			McFadden, India, 2020
			Rao, India, 2019
	Pakistan	2014	Z. Mumtaz, Pakistan, 2014
	Laos	2017	H. Catton, Laos, 2017
	Nepal	2013-2018	S. Rajbhandari, Nepal, 2018
			Bogren, Malin, Nepal, 2013
	Cambodia & Indonesia	2012-2014	Van Lerberghe, Burkina Faso, Cambodia, Morocco, Indonesia, 2014 Vieira, Bangladesh, Indonesia, Peru, 2012 Webster, Indonesia, 2013
**Latin America and Caribbean**	Peru	2012	Vieira, Bangladesh, Indonesia, Peru, 2012
**Middle East**	Jordan and Afghanistan	2014-2017	E. Speakman, Afghanistan, 2014
			P. Schwerdtle, LMICs, 2017
**Mixed (LMICs)**	-	2013-2015	Dawson AJ, 2015
			World Bank, Multiple LMICs, 2013
			Singh, Africa, Asia and Latin America/ Caribbean, 2013
			Bartlett L, 58 LMICs, 2014 Hoope-Bender, LMICs, 2014

*LMIC* low- and middle-income country

### Leadership and governance

The 33 articles that touched on leadership and governance discussed strengths and gaps. They aligned neatly with both the leadership and governance building block and the theme *national policies and administration* drawn from the review. Thirteen articles looked at the introduction of midwifery, and 18 looked at mentorship interventions. Another two looked at mentorship supporting midwives. Eight were rated high, 22 medium, and three low. The types of articles that addressed leadership and governance included intervention/ program interventions (17), retrospective country analysis (05), qualitative process review (05) and six literature reviews. Most articles on mentoring focused on local-level service delivery governance, including of managerial staff, and systems for overseeing implementation. Midwifery articles more commonly looked at national policies and guidelines. The articles broadly point to evidence-based leadership and governance that reflects ground realities being essential for midwives to practice to their full competencies. Workplace settings that are unsupportive to midwifery, a ground reality, significantly detracted from full scope midwifery and service quality, while supportive workplace settings fostered full scope practice and quality sexual, reproductive, maternal, newborn and adolescent health (SRMNAH) services. Supportive workplace settings typically involved manager engagement through various modes.

Strong leadership and clear global recommendations have helped shape national policies on midwifery and SRMNAH quality improvement [1, 9, 14, 24, 42, 76, 77] [1, 54, 61]. Global and national standards for midwives have brought stakeholders together and are an impetus to support midwifery in spite of competing interests [59]. Political will and multi-stakeholder collaboration were found essential for a quality midwifery profession [61]. Good governance of logistics and infrastructure were also identified as essential, though gaps were highlighted in adequate space for care provision, privacy for respectful care, and commodity availability [54, 56]. Gaps in midwifery leadership were also noted. Lack of leadership by midwives of the midwifery profession may contribute to reduced political will for midwifery care. Country experience shows that professions other than midwives that are positioned as leaders of the midwifery profession sometimes do not fully understand midwives’ expertise and may compete with midwives for maternity care provision roles [12].

Policies that scale up education and deploy midwives closer to communities were found to have a positive impact on service utilization and health outcomes [68]. However, gaps in both of these policy areas were also noted. First, adherence to globally recommended midwifery competencies in education programs is often not abided by in countries [54, 66, 72]. Second, World Health Organization (WHO) workforce guidelines define the number of needed health care workers per population, but not the number of midwives needed [14]. Other gaps related to deployment policy were also identified. For instance, unsupportive workplaces resulted in constraints to optimal midwifery performance in studies in both Pakistan and Bangladesh [11, 28, 74]. In these contexts, midwives either did not perform their duties, or they operated under policies that restricted them from doing so. Governance that did not take into account the inputs needed to create an environment conducive to midwifery within workplace settings was associated with lesser success. One mentoring program that reported no improvement identified restrictive national policies as a barrier [28]. Across articles, few midwives reported supportive workplace settings, and some reported humiliation, including by their direct managers [12, 39, 65, 72]. When managers were not fully involved, resistance to midwives in leadership roles, autonomous practice, and quality improvements in clinical care was found [60, 74, 78].

Conversely, local governance supporting midwife-friendly workplace settings improved midwives’ sense of competence and care quality. Webster et al. (2013) and Schuldt et al. (2020) found that midwives felt competent to provide midwife-led care in supportive workplace settings [29, 59]. Importantly, involving onsite managers to strengthen ownership of midwifery and quality SRMNAH was identified as a priority in twenty-five articles utilizing a range of methods to measure outcomes [30, 36, 60, 74, 78, 79]. Furthermore, support from managers for midwifery care and improved quality was better when on-site mentoring was present [30, 36, 60]. Seven articles found that initiating the well-known WHO quality improvement process, with a focus on SRMNAH, was an effective and easily accepted method of mentoring managers [30, 36, 49, 52, 58, 60, 69, 79]. Taneja et al. (2021) describe initial handholding support and coaching for managers as part of the quality improvement process for SRMNAH in India [79].

### Service delivery

Nearly all selected articles—47—addressed service delivery. Of these, 12 included the introduction of midwives, 29 included mentoring, and six included both (Table 7). The themes of *care quality*, *service utilization* and *health outcomes* mapped to the service delivery building block, with 39 articles addressing care quality. Due to their large number, articles addressing service delivery are discussed by theme. Of the service delivery articles, 31 were intervention evaluation, six reported retrospectively on the impact of national programs with a midwifery component (i.e., retrospective country analysis), two were qualitative process reviews, and eight were literature reviews.

### Care quality

Among the 39 articles that had a focus on care quality, nine ranked high, 28 medium, and two low. Four included the introduction of midwives, 29 included mentoring, and six included both the introduction of midwives and also mentoring. Gaps in quality education and services were noted in most articles and both introducing ICM-standard midwives as well as mentoring enabled improvements [29, 30, 34, 38, 42, 60, 64].

Studies on introducing midwives reported that ICM-standard midwives improved service quality as defined by WHO maternity care guidelines. Improvements were seen in tertiary medical centers, sub-district hospitals, and non-governmental organization (NGO) supported facilities, as well as in clinical education for nurse, doctor and midwife students [60, 64, 74, 78]. Analysis using logistic regression found that midwives significantly increased the number of women laboring in upright positions, delayed cord clamping, and immediate skin to skin contact after the birth [60, 64].

The large number of mentorship focused studies found that mentorship contributed to quality improvements in midwifery education, comprehensive and respectful SRMNAH services, and emergency obstetric and neonatal care. Studies found gains made in quality education in both classroom and clinical teaching [60, 61, 65, 66]. Midwifery educators identified that online mentoring helped them improve curriculum implementation [65]. Onsite mentoring improved teaching pedagogy, students' access to labs and libraries, and clinical teaching [64]. In addition, quality of care provided at clinical education sites improved after mentoring [60, 61, 64].

Comprehensive and respectful SRMNAH care quality showed improvements with mentoring in studies Afghanistan, Bangladesh, Rwanda, India, Nepal, Jordan, Botswana, Ethiopia, South Africa, Kenya, and Uganda [23, 44, 47, 52, 58, 62, 65, 69–71]. An analysis of program data by Save the Children and UNFPA (2021) from 47 mentored health facilities with newly deployed midwives in Bangladesh, found improved respectful communication, partograph use, upright position for birth, and companionship as well as a notable increase in health facilities receiving obstetric emergencies coming from the community [64]. Using observation data, Anderson’s et al. (2022) mixed methods observational study found that midwives in Bangladesh without mentoring made quality improvements, but, with the addition of mentoring, use of ANC cards and partograph increased significantly [60, 64].

Mentoring also contributed to better identification of high-risk pregnancies, improved diagnosis and treatment of STIs, better sterilization and cleanliness practices, and strengthened laboratory capacity to manage pregnancy and newborn-related emergencies in Rwanda [31, 38]. Research in India, South Africa, Uganda, and Rwanda found that mentoring contributed to stronger newborn care services [23, 30, 38, 39]. Despite the many documented benefits of mentoring, there is also evidence of care quality gaps remaining in programs that received mentorship. For example, Tiruneh et al. (2018) found improvements in newborn care in a study in Ethiopia, with the exception of newborn resuscitation [38] and Tripathi (2019) found little improvement in post-partum care and newborn resuscitation in India [35].

### Service availability and utilization/ health seeking behaviors

Sixteen articles touched on service availability and/or utilization [22, 24, 34, 38, 42, 50, 64, 73, 74]. Three were ranked high, 11 medium, and two low. Eight were related to introducing midwifery, five assessed mentoring interventions, and three looked at mentors who supported mentors. For the most part, the introduction of midwives was associated with increased SRMNAH service availability and utilization. In a *Lancet* article, Van Lerberghe et al. (2014) found increased facility births with multi-pronged interventions that included educating and deploying midwives [24]. Vieira et al. (2012) and Speakman et al. (2014) found greater uptake of ANC and skilled birth attendance in studies in Indonesia and Afghanistan [26, 27]. Tasnim et al. (2011) found increased ANC, facility birth, and postnatal care (PNC) following the introduction of ICM-standard midwives in Bangladesh [22]. Another study of ICM-standard midwives in Bangladesh found increases of 27%, 13% and 12% for ANC, facility birth, and PNC respectively [22, 64]. However, other Bangladesh research looking at ICM-standard midwives using a different sample of hospitals found no difference in facility births nine months after a national deployment of ICM-standard midwives [60]. Studies that found gaps in enabling policies and/or workplace settings for midwives showed fewer increased in service utilization [28, 50].

As discussed in the earlier sections, studies on mentorship interventions showed a consistent association between engaging managers in supportive workplace settings and care quality. Related to this, mentorship also influenced service availability and utilization. Two studies led by Anderson et al. (2022) and one led by Save the Children and UNFPA (2021) documented greater availability of cervical cancer screening, postpartum family planning, gender-based violence screening, and post abortion care with mentors supporting new midwives [60, 64, 74]. In research from Ethiopia, Uganda, and India, ANC and facility birth rates in their studies in Ethiopia, Uganda, and India in which mentors supported midwives. Tiruneh et al. (2018) found higher rates of care seeking for obstetric emergencies as service availability improved [38]. Namazzi et al. (2015) and Waiswa et al. (2021) found an increase of more than 20% in sick newborn care visits in Uganda [30, 56]. Stephens et al.’s (2019) study saw a more than doubling of PAC service use including associated family after a mentoring intervention focused on service quality in Tanzania [50].

### Health outcomes

Twelve articles reported health outcomes. Three ranked high, seven medium, and two low. Seven included the introduction of midwives and five had a mentoring intervention. Program interventions that were associated with improved health outcomes were thought to be the most likely to be transferable and thus were analyzed more closely to identify research methodology as well as intervention components. Ultimately, the goal of health care is to improve outcomes, and selected studies found improvements in health outcomes associated with midwife deployment [25, 28, 42].

Seven midwifery articles described improved outcomes with the introduction of midwives [24–27, 29, 43]. All were large national interventions that included many components in addition to midwives, making it difficult to ascribe attribution [24, 26, 43, 49]. One was a multi-country study evaluating the introduction of skilled birth attendants (SBAs). It had mixed results but did find decreased neonatal morbidity in Latin America, the Caribbean and partially for Asia [25]. Vieira et al. (2012), Webster et al. (2013), The World Bank (2013), and Speakman et al. (2014) found significant reductions in maternal mortality in retrospective national analyses of national midwife deployments in five countries [26, 27, 29, 42]. However, analyses of similar programs in India and Pakistan did not reduce maternal mortality [28]. The studies of projects in Indonesia and Bangladesh found that, when midwives were deployed, deaths from obstetric complications, particularly abortion, sepsis, and postpartum hemorrhage, fell over control groups of facilities that did not deploy midwives but rather used doctors and nurses in midwifery roles [22, 60, 64, 74]. Bartlet et al.’s (2014) LIST modeling exercise estimated that under even a modest scale-up, midwifery services including family planning would reduce maternal, fetal, and neonatal deaths by 34% [67].

Neonatal outcomes were inconsistent in a systematic review assessing the protective effect of SBAs on neonatal mortality in nine LMICs [25]. Where SBAs were protective in Latin America, the protection was partial in Asia, and not at all in Africa. An article from Nigeria found that SBA rate was not associated with better neonatal outcomes. Meanwhile, Viera et al. (2012) found a reduction in under 5 mortality in Brazil [26].

Four of the five mentoring articles reported improved health outcomes. All were mentoring ICM-standard nurse-midwives or midwives [52, 56, 57, 69]. In addition, all had comprehensive facility mentoring programs that visited at least twice monthly for at least three months, included managers, and strengthened data collection systems. Three were inclusive of training, two with simulation, and three provided medicine and equipment. However, certain outcomes did not improve. One study from Uganda notes declining MMR in project districts[56]. Studies from Uganda, Rwanda, and South Africa identify declining trends for stillbirths and or neonatal deaths[30, 45]. In Uganda neonatal death was reduced from 30.1 to 19.6 deaths/1,000 live births. In addition, declines in neonatal morbidity including, asphyxia were found in Kenya, Uganda, and South Africa [57].

### Health workforce

Like care quality, studies that addressed workforce comprised a relatively even split between focusing on introducing midwives and implementing mentorship programs. Seven examined midwife introduction and five mentorship; one addressed midwifery combined with mentorship. They were largely good quality with six and five rated as high and medium respectively. Two were given a low rating. The theme *experiences of midwives and their support staff* most aligned with the health workforce building block and is discussed in this section. [28–30, 60, 66]. Research quality varied and it was not always possible to discern whether attitudes were presumed or directly expressed.

Reports on midwives and other maternity staff and managers highlighted both positive and negative experiences. Some articles talked about midwives’ dissatisfaction with their workplace or feasibility to implement what was expected of them. Three studies reported midwives’ discomfort with their deployment status and the impact of those discomforts on their performance. Speakman et al. (2014) found that midwives in Afghanistan were less willing to work in military-controlled areas, stating fears about security and resistance from family [27]. Mumtaz et al. (2015) reported that newly deployed midwives in Pakistan stated difficulty in setting up private midwifery practices within rural communities, as distances made traveling prohibitive, particularly at night [28]. One study published in the *Canadian Medical Association* noted midwives' preference for positions with higher pay and not always choosing to serve the poorest [29].

Anderson et al.’s (2022) study found that midwives expressed un-elicited pride regarding their profession, particularly where midwives were enabled through mentoring. In this and other studies, midwives expressed a desire for professional autonomy, respect, and for midwifery to be a distinct profession [27, 60, 65]. In two of these studies, newly deployed ICM-standard midwives in Bangladesh expressed confidence and competence to provide quality SRMNAH services, while also expressing frustration with imposed limitations by managers and other maternity staff. Anderson et al. (2022) also found that some managers and nurses felt that midwives did not have the competence to practice autonomously or manage emergencies. Managers expressed those nurses’ felt competition with midwives and that this competition led to nurses questioning midwives’ competence.

Many articles on mentoring shared providers’, faculty’s, mentors’, and administrators’ appreciation of and knowledge gained from mentoring [52, 56, 57, 69]. Mentees in the studies in Bangladesh, Uganda, and Karnataka expressed having increased confidence and feeling happy with the mentorship and what they had learned [30, 60, 66]. Studies in India and Bangladesh observed that mentoring contributed to better teamwork among maternity staff [60]. In Laos, a mentorship program designed for newly deployed inexperienced midwives was found to be well-received by hospital administrators [31, 40]. Overall, mentoring led to positive experiences for maternity staff and maternity staff and managers were more appreciative of midwives when there was mentoring [60].

### Access to essential medicine and supplies

While it was not one of the initial themes identified, eleven articles mentioned improving essential medicines [23, 31–33, 39, 44, 45, 49, 52, 64, 68]. All examined mentoring and two discussed program interventions that introduced midwives with mentorship. Four were rated high and seven medium. Anderson et al. (2022) and Save the Children and UNFPA (2021) addressed the impact of introducing midwives on medicine availability in their studies in Bangladesh [60, 64]. In both of these articles, midwives alone did not make an impact on medicine availability. However, with mentoring, medicines became more available [60, 64]. In an example from Anderson et al.’s (2022) observational study, oxytocin and MgSo4 availability was as low as 13% in facilities without midwives or mentorship, and as high as 81% in facilities with midwives and mentors [60]. Articles on mentoring interventions in India, South Africa, Rwanda and Uganda also reported a positive impact of mentoring on availability of essential medicines [23, 30, 31, 39, 44, 45, 49, 52, 64]. Improvements may be a result of capacitating health workers and supply chain staff to activate supply chain systems. They may also be the result of improved confidence of health care providers and managers to provide the needed care and thus ensure supplies.

### Data systems

Ten articles reported on data or health information systems, also not an initial theme [23, 30–32, 36, 37, 39, 51, 56, 58, 69]. All ten looked at mentoring and reported strengthening data systems to track SRMNAH services. Articles emphasized the importance of using data to track implementation, such as a detailed clinical record which is sometimes called a case sheet [23, 31, 32, 36, 37, 56]. Namazzi et al. (2015) describes assessing the status of the patient charts and registers at baseline and then having the MOH approve file folders for inpatients to standardize record-keeping and to facilitate data availability [30]. The introduction of individual client records allows for more detailed monitoring of patient care. Synergizing SRMNAH with existing quality improvement systems included increased emphasis on and of utilization of SRMNAH data for program monitoring [36]. Taneja from India describes involving government stakeholders to build on existing data systems to ensure data-based decision making within SRMNAH [79].

### Health financing

Eight articles included a focus on health financing, which overlapped with the theme *national policies and administration* [23, 24, 26–29, 43, 68], Five related to midwifery and three included mentoring. Cost effective programs are essential in low-resource settings as even if projects are effective, sustainability is dependent on resources [56]. This review found that programs to introduce midwives and those supporting mentorship can be accomplished with minimal expenditure [23, 31]. Midwifery models were noted to be significantly less costly then obstetrician led models for care [67]. Bartlett et al. (2014) found that midwifery models were almost twice as cost-effective as obstetric models ($2,200 versus $4,200 per death averted). The introduction of midwives as well as mentoring can also be implemented within existing government systems using government employees and thus add very little additional cost. However, mentoring projects using government staff sometimes encountered constraints on availability of mentors’ time. Yet, one of the most effective mentoring projects that impacted health was implemented through existing government staff [10]. Even if project mentors are used, research from India found mentoring only increased cost by $5.60 per pregnant woman, or around $460,000 annually for eight districts, making it a cost effective intervention [23].

## Discussion

This review underscores the significance of adhering to a global midwife definition and emphasizes the importance of onsite support in creating enabling environments. The health systems building blocks served as an effective framework for interpreting the results through the lenses of its various pillars. It is worth noting that the articles focusing on midwifery, as opposed to mentorship, had fewer intervention studies and more retrospective national and qualitative process research. This research gap limits our understanding of the effective steps required for implementation of successful midwifery programs, as has been mentioned in earlier literature [5]. Among the themes explored in the midwifery literature, governance and leadership emerged as a critical first step. However, there is a pressing need for implementation research that delves into the process and impact of introducing midwives in LMICs. Drawing insights from the literature on mentoring and quality improvement holds potential for guiding countries in devising effective midwife deployment strategies [42].

One notable gap identified in this review pertains to midwife leadership [59]. The significance of midwives leading the midwifery profession has been highlighted in other literature as well [80]. Competition between professions involved in maternal health—midwifery, medicine, and nursing—for leadership roles is recognized as a hindrance to midwives fully realizing their potential. Concerns associated with non-midwives leading midwives include potential conflicting self-interest and gaps in understanding. Midwives possess unique expertise in providing quality routine SRMNAH care to essentially healthy women and newborns. If midwives are not self-governing, their distinctive vision may not be fully implemented. Therefore, more research is needed to identify best practices for promoting midwives into leadership positions.

Although the majority of articles included in this review did not explicitly address the importance of globally standard midwives, those that did emphasized its priority. The literature on skilled birth attendants emphasizes the significance of expert maternity care providers and reiterates the components of globally standard midwives. However, gaps persist in countries' adherence to global recommendations [77, 81]. Additionally, the scope of practice for midwives includes comprehensive sexual and reproductive health, as called for in the sustainable development goals. However, many non-standard midwives lack this expertise. Articles reporting changes in health outcomes consistently involved globally standard midwives, while those reporting no change often featured non-standard providers lacking basic knowledge. Further research is required to examine the impact and decision-making processes regarding the perpetuation of non-standard midwives within countries.

While all midwives provide aspects of midwifery care, this review identified significant gaps in their ability to perform to their full competencies. These gaps inevitably limit the contributions midwives can make. Enabling midwives to practice fully is particularly urgent in managing life-saving emergencies and is critical for ensuring quality respectful maternity care and comprehensive sexual and reproductive health. Frustrations regarding practice restrictions were expressed by professional midwives in this review, highlighting the importance of evidence-based leadership led by midwives themselves.

Essential medicines play a critical role in enabling environments for midwives. Notably, the findings indicate that midwives without mentoring did not impact the availability of medicine supplies. Weak supply chains are prevalent in LMICs, and stockouts of essential commodities pose significant barriers to delivering quality services. This underscores the essential role of mentoring or other forms of effective supportive supervision, particularly in this area [82]. The review suggests that mentoring involving managers and staff may help improve the availability of life-saving SRMNAH services. Further research is needed to identify the most effective methods for ensuring the availability of essential supplies and medicines through mentoring interventions.

The importance of data collection highlighted in the mentoring articles cannot be overstated. Midwives require effective monitoring of their performance to identify gaps and solve problems [83]. However, many countries still do not routinely use patient files, and the use of register books for storing patient information needed for macro data systems was noted. This review emphasizes the importance of effective gathering and utilization of information to ensure quality care and support at the micro-level. By highlighting gaps and facilitating feedback for improving care delivery, quality data at the micro-level is crucial.

The review found that evidence-based leadership and governance reflecting ground realities are essential. The 2018 WHO definition of Skilled Birth Attendant Standards emphasizes the importance of enabling environments [77]. The International Confederation of Midwives (ICM) defines an enabling environment for midwives as one that supports the necessary infrastructure, profession, and system-level integration for effective work performance [84]. Facility mentoring emerged as a critical factor in strengthening enabling environments and improving implementation quality [1, 53, 54]. Mentoring programs improve relationships between health system components and between staff and managers involved in care provision, aligning with the literature on addressing complex systems. Onsite facility mentoring, with frequent visits and involvement of all local authorities, integrated into all components of the related health system, proved to be the most effective approach. Further research is needed to determine best practices for mentoring approaches to inform program planners and policies that support workplace setting conducive to midwifery.

The facility mentoring findings in this review align closely with the WHO Quality Maternal Health Network guidelines, which emphasize on-site support, learning and sharing, measurement, community and stakeholder engagement, and program management as the key components for effective quality improvement in maternal health [76]. While WHO acknowledges the importance of management at the macro-level, this review highlights the criticality of on-site support for managers in facilitating sustainable change [85]. WHO may want to consider expanding its recommendations for managers to include micro-level support. The interventions evaluated in this review were further disaggregated by measures of success to deepen our understanding of known efficacy. Only a limited number of interventions assessed in-vivo changes in implementation. Self-reported changes or changes based on knowledge and skill were found to have limitations in effectively indicating implementation change, which reinforces the importance of on-site interventions [59, 60, 74, 76]. Mentoring programs should consider incorporating methods that evaluate observed implementation changes.

**Fig. 2:**
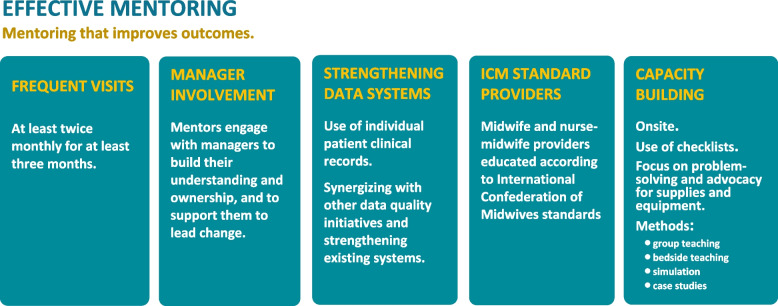
Components of mentoring that drive outcomes

Mentoring programs that demonstrated positive outcomes consisted of comprehensive packages including frequent visits, capacity building, manager involvement, and strengthening of data systems. These programs consistently mentored globally standard midwives. The high-performing programs prioritized on-site capacity building activities such as group teaching, case studies, bedside teaching, assessing and advocating for supplies and equipment, establishing and reinforcing data systems, and support for problem-solving. The use of checklists for mentors was also commonly observed in many studies (Fig. 2) [49–51].

Several limitations of this review were identified. Firstly, the included studies exhibited diversity in their approaches, delivery methods, and outcomes, resulting in considerable heterogeneity. This heterogeneity is expected, given the inclusion of studies from different countries, diverse populations, and various public health interventions, but may lead to less accurate comparisons than a more homogeneous study. Secondly, the reliance on self-reporting of outcomes as the primary outcome method of measurement may introduce some bias, either through over- or under-reporting. Third, more nuance in terms of the most effective mentorship interventions would have given more insight, specifically the efficacy of internal versus external mentorship, but although the literature did find that frequency of mentoring contacts improved outcomes, more research is needed for more specific programmatic guidance. Finally, this review might be limited by the fact that we have employed a broad categorization for 'LMIC,' which leaves gaps in understanding specific country or region contexts.

This review highlights the importance of adhering to a global midwife definition and the role of onsite support in creating enabling environments. It identifies gaps in midwife leadership and emphasizes the need for more research to promote midwives into leadership positions. The review also underscores the significance of globally standard midwives and the challenges associated with non-standard midwives. Enabling midwives to practice to their full competencies is crucial for quality care provision leading to life saving and rights upholding, and access to essential medicine plays a critical role in creating supportive environments. Effective data collection and monitoring, as well as evidence-based leadership and governance, are essential for improving midwifery care. Onsite facility mentoring emerges as a critical component of strengthening enabling environments, and more research is needed to identify best practices for mentoring approaches.

## Conclusion

Girls’ and women’s lives, and dignity depend on the availability of quality SRMNAH. To succeed in making the needed changes we need enabled, expert midwives. Midwifery literature has focused on the needed national governance and broad country understandings. There remains a need for follow up to ensure globally standard midwives are available for all girls and women. There is also a need for policy makers to include support for successful implementation. Current knowledge of health systems strengthening, and quality improvement, sheds light on the needed planning for midwives to ensure realization of their full potential. Mentoring is cost effective, and can be implemented within existing government systems. A comprehensive mentoring package inclusive of onsite capacity building of maternity staff, managers, data and procurement systems, will enable midwives to improve SRMNAH, and uphold rights for the most vulnerable.

## Data Availability

The datasets generated and/or analyzed during the current study are not publicly available due to publication restrictions from journals but are available from the corresponding author on reasonable request.

## Abbreviations

SRMNAH: Sexual, reproductive, maternal, newborn and adolescent health
LMIC: Low- and middle-income country
QI: Quality improvement
ICM: International Confederation of Midwives
SBA: Skilled birth attendants
WHO: World Health Organization
